# Health impacts of a cold wave and its economic loss assessment in China’s high-altitude city, Xining

**DOI:** 10.1186/s13690-024-01284-7

**Published:** 2024-04-18

**Authors:** Zhenxu Ning, Shuzhen He, Xinghao Liao, Chunguang Ma, Jing Wu

**Affiliations:** 1 Department of Public Health, Faculty of Medicine, Qinghai University, Xining, China; 2 Department of Public Health, Xining Centre for Disease Control and Prevention, Xining, China; 3 Xining Centre for Disease Control and Prevention, Xining, China

**Keywords:** Cold spell, Non-accidental mortality, health risk, VSL, Climate change

## Abstract

**Objective:**

Amidst climate change, extensive research has centered on the health impacts of heatwaves, yet the consequences of cold spells, particularly in cooler, higher-altitude regions, remain under-explored.

**Methods:**

Analyzing climatic data and non-accidental mortality in Xining, China’s second-highest provincial capital, from 2016 to 2020, this study defines cold spells as daily mean temperatures below the 10th, 7.5th, or 5th percentiles for 2–4 consecutive days. A time-stratified case-crossover approach and distributional lag nonlinear modeling were used to assess the link between cold spells and mortality, calculating attributable fractions (AFs) and numbers (ANs) of deaths. The study also examined the impact of cold spells over different periods and analyzed the value of a statistical life (VSL) loss in 2018, a year with frequent cold spells. Stratified analyses by sex, age, and education level were conducted.

**Results:**

A significant association was found between cold spells and non-accidental mortality, with a relative risk of 1.548 (95% CI: 1.300, 1.845). The AF was 33.48%, with an AN of 9,196 deaths during the study’s cold period. A declining trend in mortality risk was observed from 2019–2020. The 2018 VSL was approximately 2.875 billion CNY, about 1.75% of Xining’s GDP. Higher risks were noted among males, individuals aged ≥ 65, and those with lower education levels.

**Conclusion:**

The findings underscore the vulnerability and economic losses of high-altitude cities to cold spells. Implementing interventions such as improved heating, educational programs, and community support is vital for mitigating these adverse health effects.

**Supplementary Information:**

The online version contains supplementary material available at 10.1186/s13690-024-01284-7.

**Table:** 

**Text box 1. Contributions to the literature**
**•Effective addition to cold spell research at high altitudes:** This manuscript assesses the impact of cold spells in high-altitude cities on the health of populations and their impact on economic losses. It effectively expands current knowledge of the depth and extent of cold spell impacts.
**•Revealing vulnerable populations:** This study reveals that males, the elderly, and those with low levels of education are vulnerable individuals.
**•Health economic losses:** Health economic losses caused by cold spells cannot be ignored.
**•Targeted measures:** Our study highlights the importance of adopting improved heating, educational programmes and community support.

## Introduction

With climate change intensifying, various extreme weather events are becoming more frequent, such as typhoons, heatwaves, cold waves, and heavy rainfall, which have serious implications for public health. In 2019, 1.021 million deaths worldwide were caused by cold temperatures, making up 83.4% of all deaths related to non-optimal temperatures [1]. In China, 593,900 deaths were linked to non-ideal temperatures, primarily due to cold temperatures (580,800 deaths) and heatwaves (13,900 deaths), mainly stemming from cardiovascular and chronic respiratory diseases [2].This data highlight the significant influence of climate change on public health, particularly during severe weather events. Rising health burdens impact individuals, families, and economic activities. Research shows that between 2000 and 2019, severe weather events linked to climate change led to worldwide economic damages of around $2.86 trillion [3]. The significant economic loss demonstrates the wide-ranging effects of climate change on the socio-economic framework, such as rising healthcare costs, reduced agricultural productivity, and expenses related to infrastructure repairs. Therefore, we should pay more attention to extreme weather events.

Although the correlation between temperature and health has been thoroughly examined, extreme weather events have not been as widely investigated. The categorization of a cold spell, crucial for health risk assessments, frequently follows the criterion of temperatures below the first to tenth percentile for a minimum of two consecutive days [4–6]. Geographical diversity and population-specific adaptability, along with the variation in the health effects of cold spells by latitude, further increase the complexity of this research domain. In regions with lower latitudes, where average daily temperatures are typically higher, the health risks associated with cold spells may be more pronounced [7, 8]. A European study showed that the additional mortality rate was significantly higher in lower-latitude regions than in higher latitudes, with Athens showing a 2.15% increase (95% CI: 1.20, 3.10) as contrast to Finland's 0.27% (95% CI: 0.15, 0.40). Nevertheless, for regions at high latitudes, elevated altitudes, and low mean temperatures, cold spells remain a substantial health threat [9, 10]. In the instance of Xining, a high-altitude, low-temperature city in China, the health implications of cold spells could surpass those associated with heatwaves [11]. Additionally, the hypoxic conditions typical of high altitudes may contribute to secondary polycythemia, heightened blood viscosity, and increased peripheral vascular resistance, notably affecting diastolic blood pressure, thus amplifying disease risk, a situation potentially aggravated by cold spells [12].

While there had been studies exploring the extensive impacts of climate change on health and the economy, there had been little focus on the specific effects in high-altitude areas, especially the health risks and health economic losses caused by cold spells. Our study complemented existing research by analysing data from 2016 to 2020 to assess the correlation between cold spells and non-accidental deaths in Xining city. We also calculated the number of deaths caused by cold waves and the loss of VSL (the value of a statistical life). This has provided a scientific foundation for pinpointing susceptible populations in high-altitude regions and for the local government to create health strategies. We also analyzed the specific effects of cold spells at different time periods in plateau regions to gain a new perspective on the impacts of climate change.

## Material and methods

### Study sites

Xining is located in the northeastern part of the Qinghai-Tibet Plateau and has an average elevation of around 2,261 m. The climatic categorization of this region corresponds to the cold-temperate category commonly seen in high-altitude plateau areas. It is characterized by extremely cold and long winters. The annual average temperature remains at approximately 6°C, but the lowest point on the temperature scale can drop to as low as -18.9°C. In 2020, the resident population of Xining City was 2,467,965, accounting for 41.66% of Qinghai Province.

### Data collection and cold spell definitions

We systematically collected data on the daily number of deaths in Xining City from January 1, 2016, to December 31, 2020, from the Xining Centre for Disease Control and Prevention. This dataset comprised demographic variables (sex and age), educational level, cause of death, and corresponding International Classification of Diseases, Tenth Revision (ICD-10) codes. The people were put into groups based on their gender (male or female), age (0–64 years and ≥ 65 years), level of education (junior high school or less and high school or more), and cause of death, which included: non-accidental (ICD-10: A00–R99), circulatory disease (I00–I99), ischemic heart disease (IHD, I20–I25), stroke (I60–I69), respiratory disease (J00–J99), chronic obstructive pulmonary disease (COPD, J40–J47), tumor (C00–D48), and diabetes mellitus (DM, E10–E14). Meteorological parameters, including daily average temperature and humidity, were sourced from Qinghai Province's Meteorological Bureau. Five urban national monitoring stations in Xining daily recorded the concentrations of pollutants (PM_2.5_, SO_2_, CO, NO_2_, and PM_10_). In instances of air monitoring data with less than 5% missing, multiple imputation was employed for data completion [13], with an aggregate daily average from all stations representing air pollution levels. Notably, mortality and meteorological datasets were complete and exhibited logical consistency. Additionally, per capita disposable income figures for Xining City and the national average from 2015 to 2020 were extracted from the "China Statistical Yearbook 2016–2021" [14] to aid in estimating the economic impact of health-related losses.

Within this study, cold spells were defined according to previous studies as a daily mean temperature below the 5th, 7.5th, or 10th percentile lasting at least 2 to 4 consecutive days  [15].

### Statistical analysis

We conducted descriptive analysis of the main variables, primarily calculating the mean ± standard deviation (Mean ± SD), minimum (Min), median, and maximum values (Max). We also estimated associations using cold spells and mortality data for the heating period (15 October to 15 April) in Xining. Firstly, we employed a time-stratified case-crossover design. The cold spell period was defined as the case period, with control periods limited to the same day of the week (DOW) in the same month of the same year. Each case period was matched with three or four control periods before or after the case period to control for long-term trends, seasonal trends, and DOW [16].

Our study further incorporated a distributed lag non-linear model (DLNM) with conditional quasi-Poisson regression based on the time-stratified case-crossover design [17]. This was used to construct a cross-basis matrix of cold spell exposure and lag days to explore the lagged effects of cold spells on non-accidental mortality. These effects were assessed by the relative risk (RR) of non-accidental death and the 95% confidence interval (95%CI) [18]. Based on previous studies, the exposure–response relationship of cold spells in the cross-basis functions was modeled using a linear function [19], and the lag-response relationship was modeled using a natural cubic spline with 3 degrees of freedom [20], [5]. In the DLNM model, the case period (cold spell days) and control period (non-cold spell days) were assigned values of 1 and 0, respectively, allowing us to compare the exposure during the case and control periods and investigate the association between exposure and non-accidental death. Since the effects of a cold spell could last up to 2–3 weeks, we set the maximum lag at 21 days to study the lagged effects of cold spells on non-accidental mortality [21]. Additionally, we accounted for the influence of relative humidity and PM_2.5_ on pollutant concentrations in Xining by employing natural spline (ns) curves with three degrees of freedom (df) [15, 22], considering previous research findings and the prolonged heating period [23], [24].

$${\varvec{Y}}\sim {\varvec{q}}{\varvec{u}}{\varvec{a}}{\varvec{s}}{\varvec{i}}-{\varvec{P}}{\varvec{o}}{\varvec{i}}{\varvec{s}}{\varvec{s}}{\varvec{o}}{\varvec{n}}{\varvec{l}}{\varvec{o}}{\varvec{g}}({\varvec{E}}({\varvec{Y}}))=\boldsymbol{\alpha }+{\varvec{c}}{\varvec{b}}({{\varvec{C}}{\varvec{S}}}_{{\varvec{i}}},{\varvec{l}}{\varvec{a}}{\varvec{g}})+{\varvec{n}}{\varvec{s}}({\varvec{r}}{\varvec{h}},3)+{\varvec{n}}{\varvec{s}}({{\varvec{P}}{\varvec{M}}}_{2.5},3)+{\varvec{s}}{\varvec{t}}{\varvec{r}}{\varvec{a}}{\varvec{t}}{\varvec{u}}{\varvec{m}}+{\varvec{v}}{\varvec{a}}{\varvec{c}}{\varvec{a}}{\varvec{t}}{\varvec{i}}{\varvec{o}}{\varvec{n}}$$

where E(Y) represents the expected daily number of deaths; α is the intercept; cb(CS) is the cross-basis function for cold spells, used to examine the lag effects; stratum is a time-stratification variable for controlling the influence of long-term trends, seasonal variations, and other temporal factors; ns(rh, 3) is the natural cubic spline of relative humidity with 3 degrees of freedom; ns(PM_2.5_, 3) is the natural cubic spline of PM2.5 with 3 degrees of freedom; vacation is a binary variable used to control for Chinese holidays.

### Attributable fraction and attributable number

Based on the results of the association assessment, the Attributable Fraction (AF) and the Attributable Number (AN) were calculated as follows [25].

$${\varvec{A}}{\varvec{F}}=\frac{{\varvec{R}}{\varvec{R}}-1}{{\varvec{R}}{\varvec{R}}}$$

$${\varvec{A}}{\varvec{N}}={\varvec{N}}\boldsymbol{*}{\varvec{A}}{\varvec{F}}$$

where AF is the death fraction attributable to the cold spell; RR is the relative risk; AN is the number of deaths attributable to the cold spell; N is the total number of deaths.

### Calculation of VSL

To reflect the VSL associated with cold spells, the Willingness to Pay (WTP) method was used, which estimated the value of life by surveying the amount of money people were willing to pay to reduce the risk of death [26–28]. This study referenced a WTP study conducted in 74 Chinese cities [29] and adjusted it according to the per capita annual income of Xining during the study period to obtain the statistical value of life for the residents.

$${{\varvec{V}}{\varvec{S}}{\varvec{L}}}_{{\varvec{y}}}={\left({{\varvec{V}}{\varvec{S}}{\varvec{L}}}_{{\varvec{b}}{\varvec{a}}{\varvec{s}}{\varvec{e}}}\boldsymbol{*}\frac{{{\varvec{I}}{\varvec{N}}{\varvec{C}}{\varvec{O}}{\varvec{M}}{\varvec{E}}}_{{\varvec{y}}}}{{{\varvec{I}}{\varvec{N}}{\varvec{C}}{\varvec{O}}{\varvec{M}}{\varvec{E}}}_{{\varvec{b}}{\varvec{a}}{\varvec{s}}{\varvec{e}}}}\right)}^{{\varvec{e}}}$$

where ${VSL}_{y}$ presents the statistical value of life for the study subjects; ${VSL}_{base}$ is the statistical value of life for China in 2015 as estimated by the cited literature; ${INCOME}_{base}$ is the per capita annual income for China in 2015; ${INCOME}_{y}$ is the per capita annual income for the study subjects; and $e$ is the income elasticity coefficient, assumed to be 1. The number of deaths attributable to cold spells, multiplied by the statistical value of life, equals the health economic loss related to cold spell-associated deaths.

### Sensitivity analysis

We used sensitivity analysis to test the stability of the model. To verify the associations and make comparisons, we adjusted the lag dimension of cold spells and the lag dimension of relative humidity from 3 to 6 [30]. The lag days for cold spells were adjusted from 0–21 days to 0–30 days. Furthermore, we incorporated single air pollutants (NO_2_, CO, and SO_2_) as well as combined air pollutants (PM_2.5_&NO_2_, PM_2.5_&SO_2_, and PM_2.5_&NO_2_&SO_2_) into the model separately to explore the impact of confounding factors on the results [31], [32].

This study primarily used R software (version 4.3.1) for statistical analysis, with time series analysis constructed using the "DLNM" and "splines" packages.

## Results

Table 1 and Fig. A 1 present a descriptive analysis of meteorological variables, air pollutants, and counts of non-accidental deaths. During the study period, the daily average temperature and daily average relative humidity in Xining city were 6.4 ± 9.2 (°C) and 57.5 ± 15 (%), respectively. The daily average concentrations of PM_2.5_, SO_2_, NO_2_, and CO were 41.1 ± 27 μg/m^3^, 20.4 ± 13.9 μg/m^3^, 39.9 ± 16.9 μg/m^3^, and 1.4 ± 0.8 mg/m^3^, respectively. From 2016 to 2020, total deaths from non-accidental causes amounted to 48,756, with 26,237 being attributed to circulatory disease, 9,650 to IHD, 10,235 to stroke, 6,090 to respiratory disease, 4,803 to COPD, 11,716 to tumor,and 2,057 to DM. Figure A 2 shows that a low to moderate correlation (*p* < 0.05) was observed between the daily average temperature and other variables. Among all the variables involved in the study, the correlation between PM_2.5_ and CO was relatively strong, with a correlation coefficient greater than 0.7, while the correlation between SO_2_ and relative humidity was the lowest.

**Table 1:** Daily meteorological, pollutant, and mortality data for Xining City from 2016 to 2019

	Counts	Mean ± SD	Min	Median	Max
**Meteorological variables**					
Daily mean temperature (°C)	/	6.4 ± 9.2	-16.2	7.3	25.6
Daily mean relative humidity (%)	/	57.5 ± 15.8	15	58	94
**Air pollutants**					
PM_2.5_ (μg/m^3^)	/	41.1 ± 27.0	4	33	213
SO_2_ (μg/m^3^)	/	20.4 ± 13.9	1	17	133
NO_2_ (μg/m^3^)	/	39.9 ± 16.9	1	38	110
CO(mg/m^3^)	/	1.4 ± 0.8	0.2	1.2	6.1
**Deaths**					
Non-accidental	48,756	26.7 ± 9.4	1	25	74
Circulatory	26,237	14.4 ± 6.9	1	13	52
IHD	9,650	5.3 ± 2.7	0	5	21
Stroke	10,235	5.6 ± 2.9	0	5	19
Respiratory	6,700	3.6 ± 2.1	0	3	16
COPD	4,803	2.6 ± 0.8	0	2	16
Tumor	11,716	6.4 ± 2.8	0	6	19
DM	2,057	1.1 ± 1.1	0	1	6

Figure A 3 depicts the demographic profile of Xining City based on the 2020 census. The resident population amounted to 2,467,965 individuals. Gender-wise, males constituted 50.91%, while females represented 49.09% of the total population. In terms of education, there were 1,600,394 people with a junior high school education or less and 867,571 with a high school education or more. An overview of the age composition highlights that the population aged 0–64 years encompassed 2,218,296 people, and those aged ≥ 65 years encompassed 249,669 people, accounting for 10.12% of the total population. Compared to previous figures, there has been a rise of 2.60 percentage points in the proportion of people aged ≥ 65 years, thereby indicating an inclination towards an aging demographic.

**Fig. 1:**
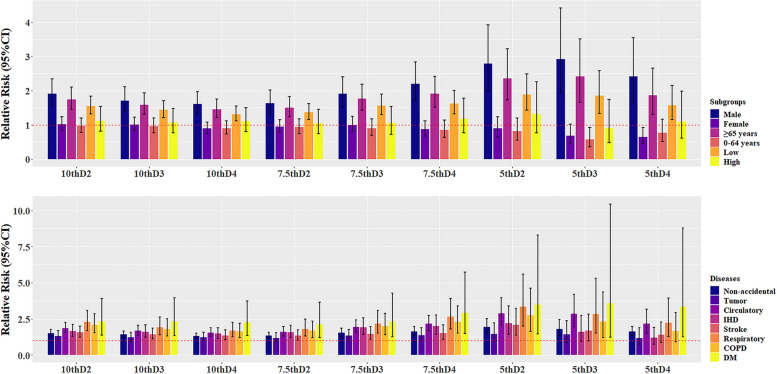
Cumulative effect of cold spells on mortality for days 0–21 under the nine cold spell definitions, stratified by sex, age, level of education and cause (Non-accidental, Circulatory disease, IHD, Stroke, Respiratory disease, COPD, Tumor, and Diabetes mellitus). Cold spells were defined by the percentile temperature threshold (5th, 7.5th, or 10th percentiles) and by the number of consecutive days below the threshold (2–4 days, indicated by D2, D3, D4). “Low” represents junior high school and below, and “High” represents high school and higher

Table A 1 summarizes the impact of different definitions of cold spells on non-accidental mortality among residents of Xining city during the study period and the model's generalized cross-validation (GCV). The number of cold spell days and the relative risk values were influenced by the threshold and the duration of the temperature. When defining the cold spell as a daily average temperature below the 10th percentile lasting at least two days (10th-D2), there were 168 cold spell days identified, with the relative risk (RR) for non-accidental mortality of 1.519 (95% CI: 1.308, 1.765), indicating an increased death risk of 51.9% (95% CI: 30.8, 76.5). Furthermore, Fig. 1 and Fig. A 4 illustrate the RR for the period of 0–21 days and the day of the cold spell under nine different cold spell definitions, which were categorized according to the cause of death and individual characteristics. These factors negatively impacted non-accidental deaths within the population, and the confidence intervals for the effect estimates widen as the definition of cold spells became stricter. Based on these preliminary results and referencing previous studies, we selected the 7th-D3 definition, which had the smallest GCV, as the optimal definition of the cold spell [15], [19].

**Fig. 2:**
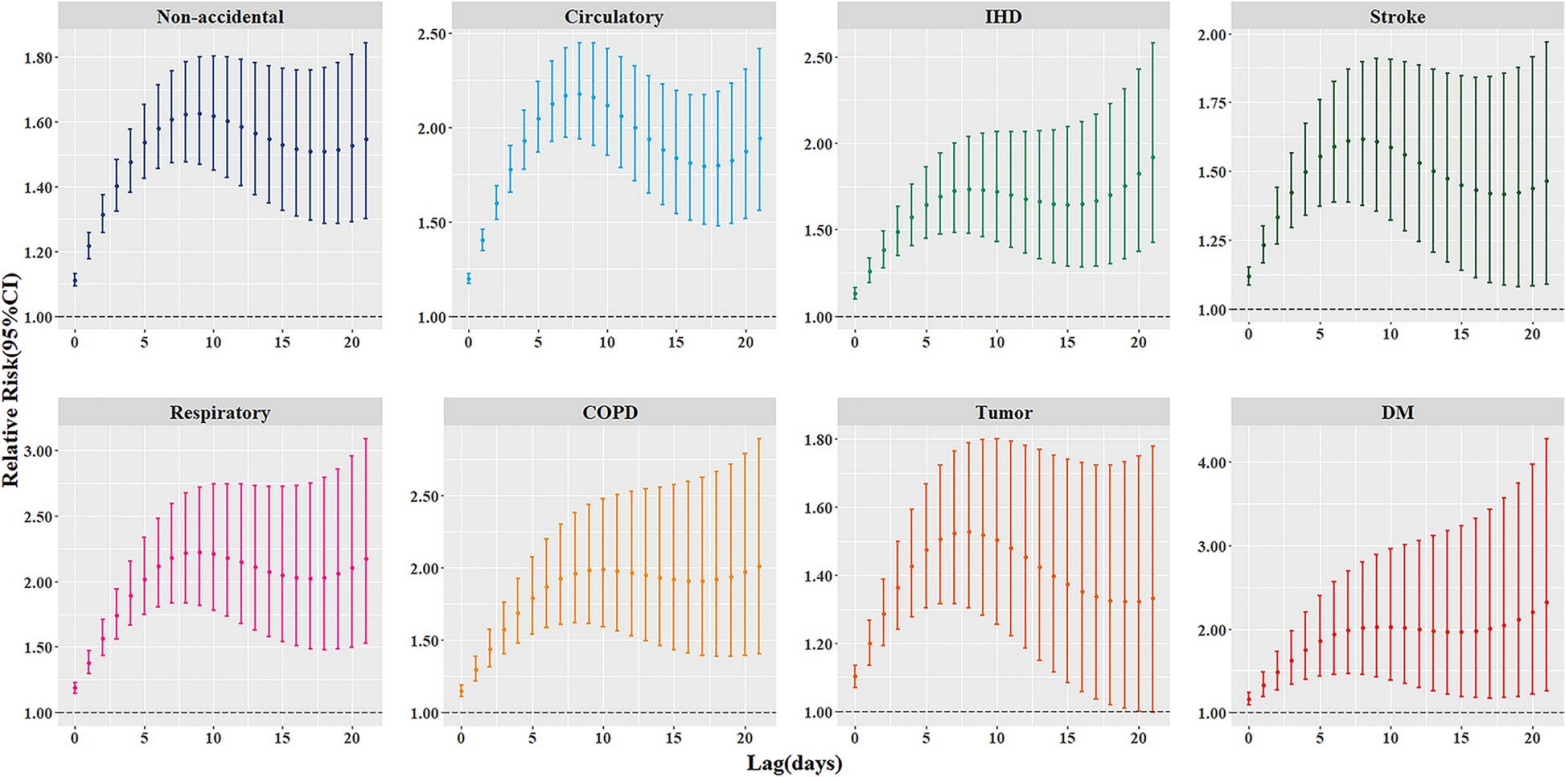
Cumulative lagged effects of the optimal cold spell on different specific causes of death (Non-accidental, Circulatory disease, IHD, Stroke, Respiratory disease, COPD, Tumor, and Diabetes mellitus) across the lags of 0–21 days

Figure 2 and Fig. A 5 show the single-day and cumulative lagged effects of the optimal cold spell on specific cause of death categories for residents of Xining between 2016 and 2020. Information on the specific estimates can be found in Table A 2. In the analysis of single-day lagged effects, this study found significant correlations between cold spell events and non-accidental mortality among residents. For non-accidental, stroke, respiratory disease, COPD, tumor, and diabetes, all these disease deaths had the highest risk of dying on the day of the cold spell event, with RRs of 1.11 (95% CI: 1.092, 1.130), 1.118 (1.086, 1.152), 1.187 (1.148, 1.228), 1.148 (1.109, 1.189), 1.103 (1.071, 1.135), and 1.163 (1.095, 1.235). Notably, they were no longer statistically significant after lag day 8. For deaths from circulatory disease and IHD, the RR was highest on the day of the cold spell event, with RRs of 1.200 (95% CI: 1.174, 1.226) and 1.133 (1.100, 1.167), respectively, and was no longer statistically significant after lag day 8, with the effect reappearing on lag day 20.

Regarding the analysis of cumulative lag effects, there were significant cumulative lag effects between cold spells and cause-specific diseases. These effects were consistently significant over the cumulative lag period of 0–21 days, with similar trends. The longer the cumulative lag, the wider the confidence interval. The highest value for the total effect of non-accidental deaths was 1.626 (95% CI: 1.469, 1.800) for the cumulative lag period of 0–9 days. It is worth noting that the cumulative RR for respiratory deaths was slightly higher than that for circulatory deaths: 2.223 (95% CI: 1.818, 2.719) and 2.179 (95% CI: 1.939, 2.448), respectively. These results highlight the potential risk of cold spells on the health of the population, with particularly significant effects during the cumulative lag period.

Figure A 6 shows the cumulative lagged effects of the cold spell on specific causes of death for different residents of Xining City over two periods, from 2016 to 2018 and from 2019 to 2020. Comparing these two periods, we could observe a general decreasing trend in the cumulative relative risk of causes of death, except for the RR of circulatory disease and IHD deaths, which increased in days 0–21 of the cumulative lag.

Table 2 summarises the impact of cumulative lag effects under the most suitable cold spell conditions, stratified by sex, age, and level of education. Males, individuals aged ≥ 65 years, and those with a lower level of education were more vulnerable to the effects of cold spells, with similar cumulative lag trends. The maximum cumulative RR for these groups were 2.084 (95%CI:1.827,2.377),1.932 (1.723,2.165),1.738 (1.569,1.925), respectively. Females, individuals 0–64 years, and those with a higher level of education did not experience a significant risk of death due to cold spells. These results emphasize the differences in the impact of cold spells with regard to sex, age, and education level.

**Table 2:** Relative risks of non-accidental mortality due to cold spells stratified by sex, age, and educational level.(“Low” represents junior high school and below, and “High” represents high school and higher)

Subgoups	lag0	lag0-7	lag0-14	lag0-21
**Sex**				
Male	1.195(1.168,1.222)	2.102(1.878,2.353)	1.802(1.511,2.150)	1.904(1.514,2.395)
Female	1.005(0.982,1.028)	1.063(0.948,1.192)	1.106(0.929,1.317)	1.011(0.808,1.265)
**Age(years)**				
≥ 65	1.160(1.137,1.185)	1.905(1.716,2.116)	1.727(1.465,2.035)	1.757(1.420,2.173)
0–64	1.008(0.980,1.035)	1.020(0.891,1.167)	0.981(0.799,1.204)	0.931(0.715,1.211)
**Education level**				
Low	1.136(1.115,1.157)	1.727(1.572,1.897)	1.566(1.353,1.813)	1.564(1.295,1.889)
High	1.022(0.984,1.061)	1.097(0.909,1.324)	1.078(0.809,1.437)	1.079(0.741,1.571)

**Fig. 3:**
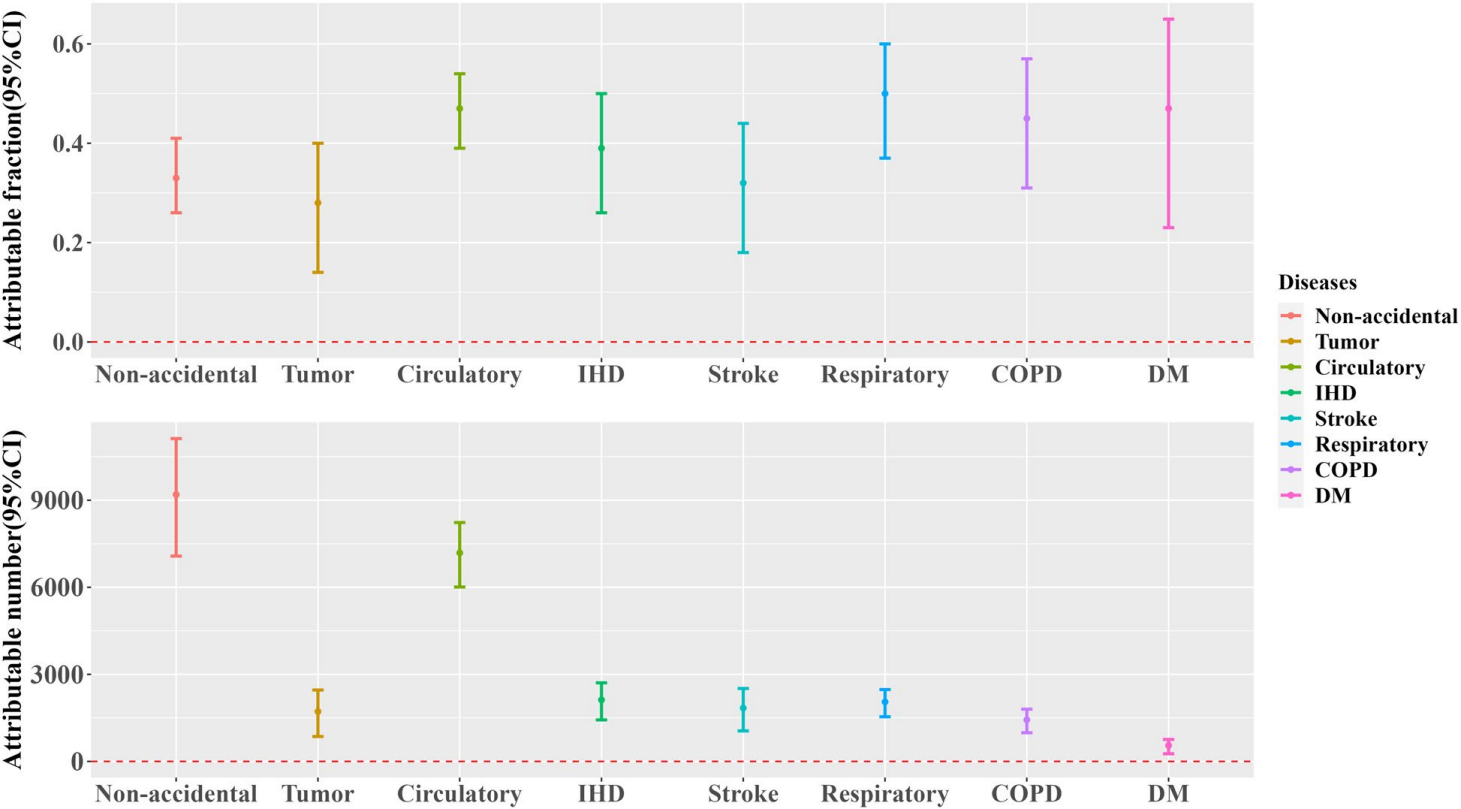
Attributable proportions and attributable numbers for specific causes of death under optimal cold spell. (Non-accidental, Circulatory disease, IHD, Stroke, Respiratory disease, COPD, Tumor, and Diabetes mellitus)

**Fig. 4:**
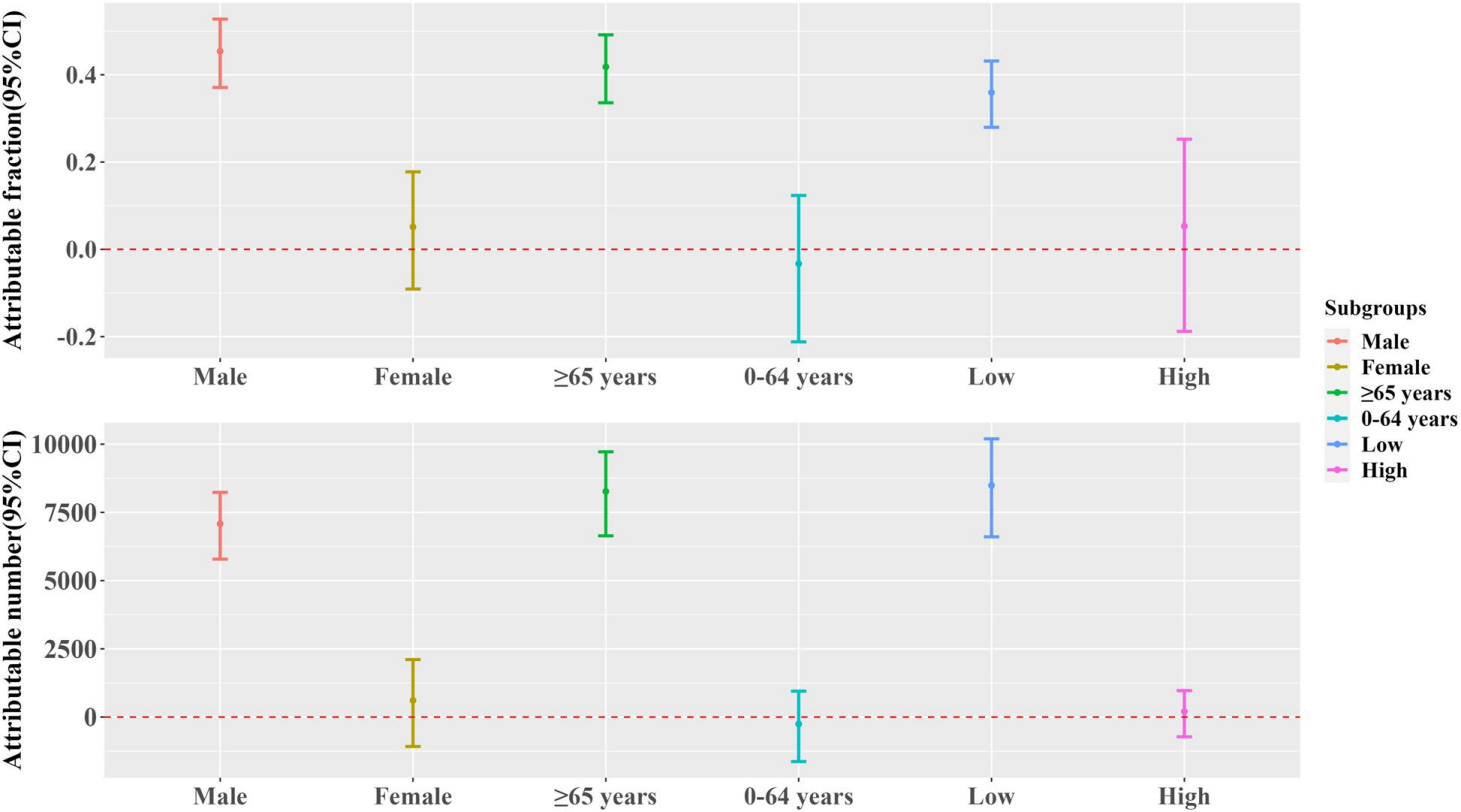
Attributable fraction and attributable number of the optimal cold spell, stratified by sex, age, and level of education

Figures 3 and 4 show the attributable fraction and attributable number for different diseases and by gender, age, and education level, respectively. The analysis revealed that cold spells were associated with 9,196 non-accidental deaths, corresponding to an attributable fraction (AF%) of 33.48 (95%CI: 25.76, 40.51). Additionally, the study identified substantial variations in cold spell-related mortality among different diseases and demographic segments. It is worth mentioning that deaths resulting from respiratory illnesses due to cold weather had an AF of 49.65 (95% CI: 37.23, 59.52), slightly surpassing those of circulatory ailments at 46.94 (95% CI: 39.27, 53.77). This resulted in excess deaths of 2,034 and 7,253, respectively. The number of deaths attributable to IHD, stroke, COPD, tumors, and DM was 2,120, 1,838, 1,433, 1,720, and 551, respectively. For different population characteristics, there were 7,060 excess deaths for males, 8,185 for people aged ≥ 65 years, and 8,452 for those with lower educational levels. In these groups, AF increased significantly to 45.40 (95%CI: 37.09, 52.76), 41.81 (33.59, 49.15) and 35.94 (27.96, 43.16), respectively. No statistically significant differences were observed in this study for females, people aged 0–64 years, and individuals with higher levels of education.

Table A 3 lists the number of days with cold spells for each year, with 2018 having the highest number of cold spell days. Therefore, we chose to base our analysis of the health economic losses caused by cold spells on the year 2018. According to data from the China Statistical Yearbook, the per capita disposable income in China was about 21,966 CNY in 2015, while in Xining City it was about 25,926 CNY in 2018. Through calculations, we have obtained the following results: The VSL caused by all non-accidental deaths due to cold spells in Xining City was approximately 2.875 billion CNY (95% CI: 1.152, 4.318). Among them, the VSL caused by circulatory disease and respiratory disease were 2.334 billion CNY (95% CI: 1.259, 3.206) and 0.677 billion CNY (95% CI: 0.160, 1.042), respectively. The VSL for all non-accidental deaths due to cold spells accounts for 1.75% of Xining City's GDP. Compared to cities in the eastern and southern parts of China, the health economic loss in Xining city is relatively low, which is mainly related to the city's centralised heating and strong adaptability to low temperatures.

Figure A 7 sensitivity analysis shows that with the changes in the dimensions of cold spell lag, the number of lag days, and the degrees of freedom for relative humidity in the model, the amplitude of all the changes was small. The results remained consistent even when considering single air pollutants and combinations of pollutants. These sensitivity analysis results strengthen the robustness and reliability of our research findings.

## Discussion

Our study investigated the correlation between cold spells and non-accidental mortality in the high-altitude urban area of Xining. Additionally, the study evaluated the VSLs by analysing the number of deaths attributed to cold spells in conjunction with per capita income. The findings indicate that instances of cold spells had a statistically significant adverse effect on non-accidental mortality.

In the single-day lag structure, this study found that cold spell events had a significant impact on non-accidental deaths among residents of Xining city on the day of the event. This result was similar to findings from studies conducted in China, where the RR of mortality on the day of a cold spell was 1.01 (95% CI: 1.00, 1.03) [20]. The cumulative lag model revealed a significant positive association between cold spells and non-accidental mortality among the population. The risk of non-accidental deaths in Xining during cold spells was close to the estimated value for capital cities in China using data from 2007 to 2013 (RR for Xining: 1.44; 95%CI: 1.15, 1.81; RR for all of China: 1.55; 95%CI: 1.40, 1.70). The estimated mortality rates for circulatory and respiratory diseases were similar (RR: 1.69; 95%CI: 1.48, 1.89; RR: 1.88; 95%CI: 1.65, 2.11) [20]. The risk of non-accidental mortality in Xining was slightly higher compared to earlier studies conducted between 2007 and 2013, possibly due to the aging population. In our study, non-accidental deaths among the elderly accounted for about 70% of total deaths. According to the 2020 census in Xining, the proportion of the population aged ≥ 65 years increased by 2.60 percentage points. This indicates that although the immediate impact of cold spells is consistent across different regions, the long-term cumulative effects may vary due to regional characteristics. In comparison to cities located at higher latitudes and experiencing lower average temperatures, like Murmansk (RR: 1.12,95%CI: 1.07, 1.17) [33], Xining had a greater risk of non-accidental death caused by cold spells. This may reflect the regional differences in the impact of cold spells due to geographic and climatic conditions, indicating that cold spells also have a significant impact on the health of residents in cities with long cold periods. The cumulative lag effect in our study lasted for 2–3 weeks, consistent with previous studies [11, 15]. Experiments also demonstrated that the human body requires approximately 2–3 weeks to adjust to cold conditions [34]. Comparisons of cold spells at different times showed that over time, the RR of death generally decreased, which may be related to global warming and a reduction in the occurrence of cold spells [35, 36]. Nevertheless, despite global warming, cold spell events may still pose significant health risks in this century [37], [38]. This finding emphasizes the importance of continued attention to the impact of extreme weather events on public health in the context of climate change and suggests the need to consider the effects of regional characteristics and demographic changes on health risk assessments.

Stratified analysis shows that the impact of cold spells varies among different populations, with males, older age groups, and individuals with lower levels of education exhibiting greater sensitivity, while no statistically significant differences were observed in other subgroups. Further research indicates that the risk of non-accidental death among males is slightly higher than that among females, consistent with previous findings [28, 39]. A study conducted in Qingyang, a city in close proximity to Xining [40], likewise revealed that males have a greater susceptibility to adverse effects in low temperature environments. This difference may be attributed to men's higher involvement in outside labour, the greater indoor-outdoor temperature difference, and demographic factors. It has been suggested that significant disparities between indoor heating temperatures and outdoor non-heating temperatures could result in higher mortality rates [41]. Significantly, Xining's heating period lasts for half a year, which may further amplify the impact of temperature differences on mortality. Moreover, the elderly population was at a higher risk of death, a result that corresponds with other investigations [42, 43], [44]. Some research indicates that as age increases, the skin's reflective vascular constriction response to cold weather may become excessive or insufficient, potentially having a pathological impact on individuals already at risk of cardiovascular events [45]. Individuals with lower educational levels present a much higher mortality risk than those with higher educational levels, a result that aligns with previous research [45], [20]. This suggests that the impact of cold spells on residents may be associated with factors such as low income, inadequate household hygiene, and insufficient heating infrastructure [28, 46]. However, a study in Shanghai did not find a statistical correlation between education level and temperature change [47]. This disparity could be attributed to the greater percentage of residents with lower educational level in Xining, constituting approximately 65% of the overall population.

From 2016 to 2020, the total number of non-accidental deaths in Xining city attributed to cold spells was 9,196, with an attributable fraction (AF%) of 33.48 (95%CI: 25.76, 40.51). This result (Xining's CER = 54.7%) is close to the previously reported excess mortality rates during cold spells in Central China (57.1%) and East China (55.5%) [5]. In comparison with a study of the attributable fractions for cold-related mortality in several Chinese cities (Xining's AF: 20.28,95%CI: 6.52, 34.04) [41], the attributable fraction for mortality due to cold spells in Xining is significantly higher. Compared with other Chinese cities, Xining's attributable fraction is slightly higher than Shijiazhuang's 20.37 (95%CI: 13.10, 27.64) and Nanjing's 20.75 (95%CI: 6.63, 34.87) [48], which may be associated with the use of heating period data and regional differences in this study. This is below the findings of a study on cold spells and influenza in the United States (Wyoming: AF = 47.91%, 95%CI: 36.13%, 57.17%) [49].

In 2018, the VSL caused by non-accidental total deaths due to cold spells in Xining city was approximately 2.875 billion CNY (95%CI: 1.152, 4.318), accounting for 1.75% of the city's GDP. This loss was significantly different from other studies conducted in the same city [50, 51], where the VSL for Xining city was estimated at 3.12 and 6.524 billion CNY, respectively. The differences mainly arose from estimation methods, the number of deaths, the study period, and the regions involved. The health and economic loss in Xining city due to cold spells was relatively lower compared to regions like Beijing, Tianjin, Guangdong, Shanghai, and others in eastern and southern China [50]. The difference could be attributed to the centralised heating system in Xining, which offered residents more consistent and extensive heating, thereby lowering health risks in cold weather. The per capita income and population size of Xining City were also factors that affected the regional disparities in health and economic losses. The economic losses to health were relatively low in the case of cold waves compared to heat waves. Studies in France have shown an economic impact of 25.5 billion euros for the specific health effects of heat waves, with mortality amounting to 23.2 billion euros [26]. This could have something to do with taking active steps to stay warm when the cold spell hits. These emphasised the significant role of region-specific socio-economic conditions and public policies in reducing the impacts of extreme weather events. This provided a scientific foundation for local governments to create specific health policies and interventions to protect vulnerable groups.

Current research [52], [53] increasingly indicates that cold spells have a negative impact on public health, with prolonged exposure to cold conditions potentially increasing the risk of heart attacks, malignant cardiac arrhythmias, coronary artery complications, and cerebral thrombosis [54, 55]. However, there is a scarcity of research in high-altitude areas, where large temperature variations, extreme weather events (such as blizzards and strong winds), and significant differences in air pressure make residents more susceptible to the effects of cold spell events. It has also been suggested [56, 57] that living at high altitude may have a protective effect on coronary heart disease mortality, and studies in Switzerland [58] have suggested that this may be related to climate change. Confounding factors contributing to this association may also include the role of solar radiation and vitamin D [59, 60]. However, the onset of the cold spell may increase the risk [61], [62]. This study effectively addresses the gaps in research on high-altitude areas and provides crucial information for a better understanding of the health risks faced by these regions in the context of climate change.

This study has certain limitations. Firstly, meteorological and pollution data mainly came from monitoring stations, not individual exposure data, which may lead to exposure misclassification. Secondly, as an ecological study, it is difficult to control for individual risk factors. Thirdly, the population size of the study area is small, with significant differences in educational levels, and the non-accidental death data are relatively limited compared to other areas, which could all affect the results of the study.

Based on this study's assessment of the vulnerability and the VSL of high-altitude cities during cold spell events, we suggest that governments, when developing comprehensive cold spell event management policies, pay special attention to vulnerable groups such as the elderly, males, and those with lower levels of education. For these groups, the government should design and implement specific intervention measures, including but not limited to providing easily accessible heating facilities and cold-weather materials for the elderly, conducting targeted health risk education and awareness activities for men, and setting up information distribution points and educational seminars for residents with lower levels of education to ensure that information on cold spell warnings and health protection measures is widely disseminated. Furthermore, considering the unique geographical and climatic conditions of high-altitude cities, it is recommended that the government strengthen infrastructure construction, such as improving and maintaining heating systems to ensure their continuous operation during cold spells and establishing multi-level warning systems, including at the community, school, and family levels, to achieve a rapid response to cold spell events. The establishment of community support networks, especially the organisation of volunteer teams, can provide timely help and support to vulnerable groups during cold wave events, including food, warm clothing, and necessary medical care. By taking these measures to improve the quality of life for residents of high-altitude cities, community resilience can also be enhanced, enabling better coping with future climate change and extreme weather events.

In this study, cold spells significantly increased non-accidental deaths in Xining city, especially on the day of the cold spell and for 2–3 weeks afterward. The analysis shows that cold spells affect vulnerable groups, like the elderly. Although death risk has decreased, cold spells still affect public health, emphasising the need for targeted interventions to protect vulnerable groups.

### Supplementary Information

**Supplementary Materials1.**

## Data Availability

As the data on the cause of death contains a significant amount of personal information concerning the deceased and their families, it cannot be published. Please direct any additional queries to the corresponding author.
