# Supplementary material for: Health impacts of a cold wave and its economic loss assessment in China’s high-altitude city, Xining
Source: Arch Public Health. 2024 Apr 18;82:52. doi: 10.1186/s13690-024-01284-7 (PMC11025205; doi:10.1186/s13690-024-01284-7)

Supplementary Material

# Supplementary Tables

**Table A1** Number of days, model GCV, and the relative risks (RR) under different definitions of cold spells. Cold spells were defined by the percentile temperature threshold (5th, 7.5th, or 10th percentiles) and by the number of consecutive days below the threshold (2-4 days, indicated by D2, D3, D4).

| cold spell definition | Duration (d) | Cold spell days (d) | GCV | RR(95%CI) |
| --- | --- | --- | --- | --- |
| 10^th^-D2 | 2 | 168 | 2.7019 | 1.519(1.308,1.765) |
| 10^th^-D3 | 3 | 157 | 2.7484 | 1.422(1.216,1.663) |
| 10^th^-D4 | 4 | 136 | 2.7697 | 1.316(1.134,1.526) |
| 7.5^th^-D2 | 2 | 137 | 2.6738 | 1.353(1.156,1.584) |
| 7.5^th^-D3 | 3 | 122 | **2.6217** | 1.548(1.300,1.845) |
| 7.5^th^-D4 | 4 | 99 | 2.6565 | 1.634(1.346,1.984) |
| 5^th^-D2 | 2 | 81 | 2.6998 | 1.948(1.512,2.509) |
| 5^th^-D3 | 3 | 66 | 2.7315 | 1.813(1.331,2.470) |
| 5^th^-D4 | 4 | 45 | 2.9541 | 1.420(1.060,1.901) |

**Table A2** The estimates of the optimal cold spell on different specific causes of death across the lags of 0–21 days.

| Lag structure | RR (95%CI) | | | | | | | | |
| --- | --- | --- | --- | --- | --- | --- | --- | --- | --- |
|  |  | Non-accidental | Circulatory | IHD | Stroke | Respiratory | COPD | Tumor | DM |
| Single-day lag | 0 | 1.111(1.092,1.130) | 1.200(1.174,1.226) | 1.133(1.100,1.167) | 1.118(1.086,1.152) | 1.187(1.148,1.228) | 1.148(1.109,1.189) | 1.103(1.071,1.135) | 1.163(1.095,1.235) |
|  | 7 | 1.018(1.008,1.029) | 1.020(1.007,1.033) | 1.017(1.000,1.035) | 1.013(0.995,1.031) | 1.031(1.010,1.052) | 1.031(1.009,1.053) | 1.012(0.994,1.029) | 1.028(0.991,1.066) |
|  | 14 | 0.988(0.978,0.998) | 0.972(0.960,0.984) | 0.992(0.976,1.009) | 0.981(0.965,0.998) | 0.983(0.964,1.003) | 0.992(0.972,1.012) | 0.981(0.965,0.997) | 0.995(0.961,1.030) |
|  | 21 | 1.014(0.996,1.032) | 1.038(1.015,1.061) | 1.051(1.021,1.083) | 1.018(0.988,1.048) | 1.031(0.996,1.068) | 1.022(0.987,1.058) | 1.006(0.977,1.037) | 1.054(0.992,1.119) |
| Multi-day lag | 0 | 1.111(1.092,1.130) | 1.200(1.174,1.226) | 1.133(1.100,1.167) | 1.118(1.086,1.152) | 1.187(1.148,1.228) | 1.148(1.109,1.189) | 1.103(1.071,1.135) | 1.163(1.095,1.235) |
|  | 0-7 | 1.609(1.473,1.757) | 1.883(1.591,2.229) | 1.723(1.483,2.001) | 1.612(1.388,1.871) | 2.180(1.832,2.594) | 1.925(1.610,2.301) | 1.523(1.316,1.764) | 1.987(1.464,2.698) |
|  | 0-14 | 1.547(1.349,1.773) | 1.942(1.561,2.415) | 1.648(1.307,2.078) | 1.473(1.169,1.856) | 2.074(1.579,2.724) | 1.931(1.459,2.556) | 1.398(1.115,1.753) | 1.966(1.219,3.171) |
|  | 0-21 | 1.548(1.300,1.845) | 1.925(1.548,2.394) | 1.920(1.428,2.581) | 1.464(1.089,1.968) | 2.171(1.527,3.087) | 2.013(1.403,2.888) | 1.332(0.997,1.780) | 2.321(1.260,4.275) |

**Table A3** Number of days under optimal definition of cold spells.

| Date | Cold spell days(d) |
| --- | --- |
| 2016 | 32 |
| 2017 | 14 |
| 2018 | **44** |
| 2019 | 20 |
| 2020 | 12 |

1. **Supplementary Figures**

**Figure A1** Time series plot of various causes of death in Xining from 2016 to 2020 (Non-accidental).

**
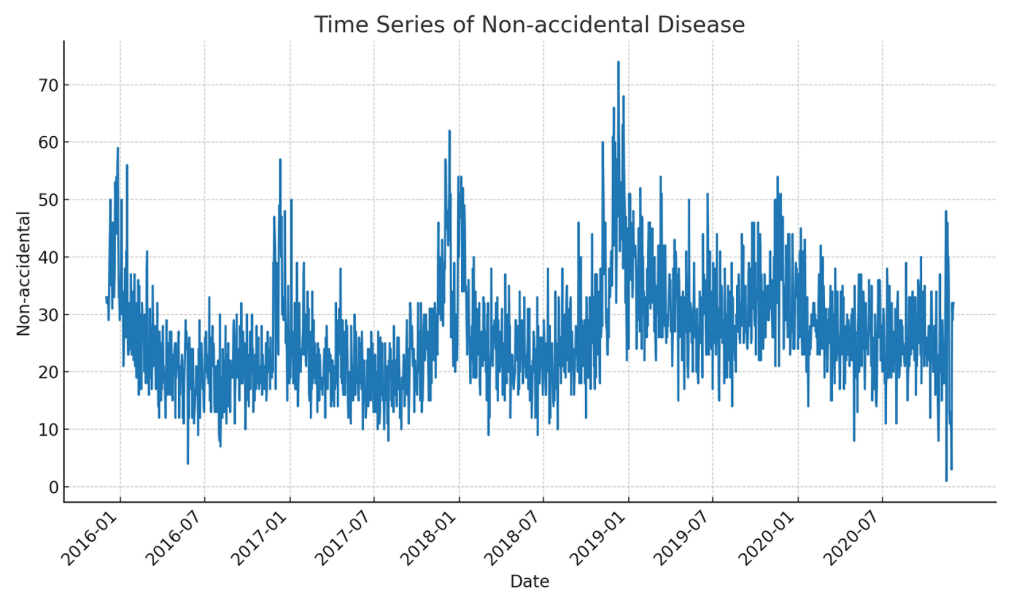
**

**Figure A2** Spearman correlation between air pollutants and meteorological factors in 2016-2020 of Xining City, Qinghai Province, China. (*P < 0.05)


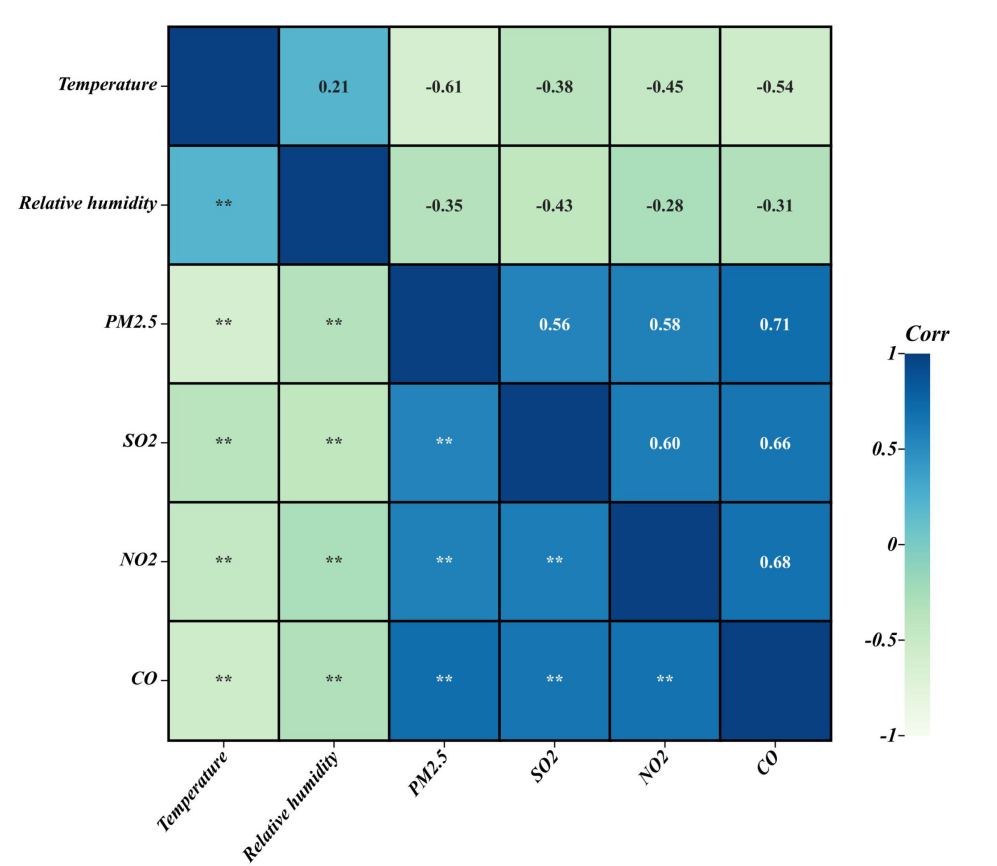


**Figure A3** Population distribution in Xining City, Qinghai Province, China in 2020. (“Low” represents junior high school and below, and “High” represents high school and higher)





**Figure A4** Single-day effect of cold spells on mortality for days 0–21 under the nine cold spell definitions, stratified by cause, sex, age, and level of educatio (Non-accidental, Circulatory disease, IHD, Stroke, Respiratory disease, COPD, Tumor, and Diabetes mellitus)n. Cold spells were defined by the percentile temperature threshold (5th, 7.5th, or 10th percentiles) and by the number of consecutive days below the threshold (2-4 days, indicated by D2, D3, D4).


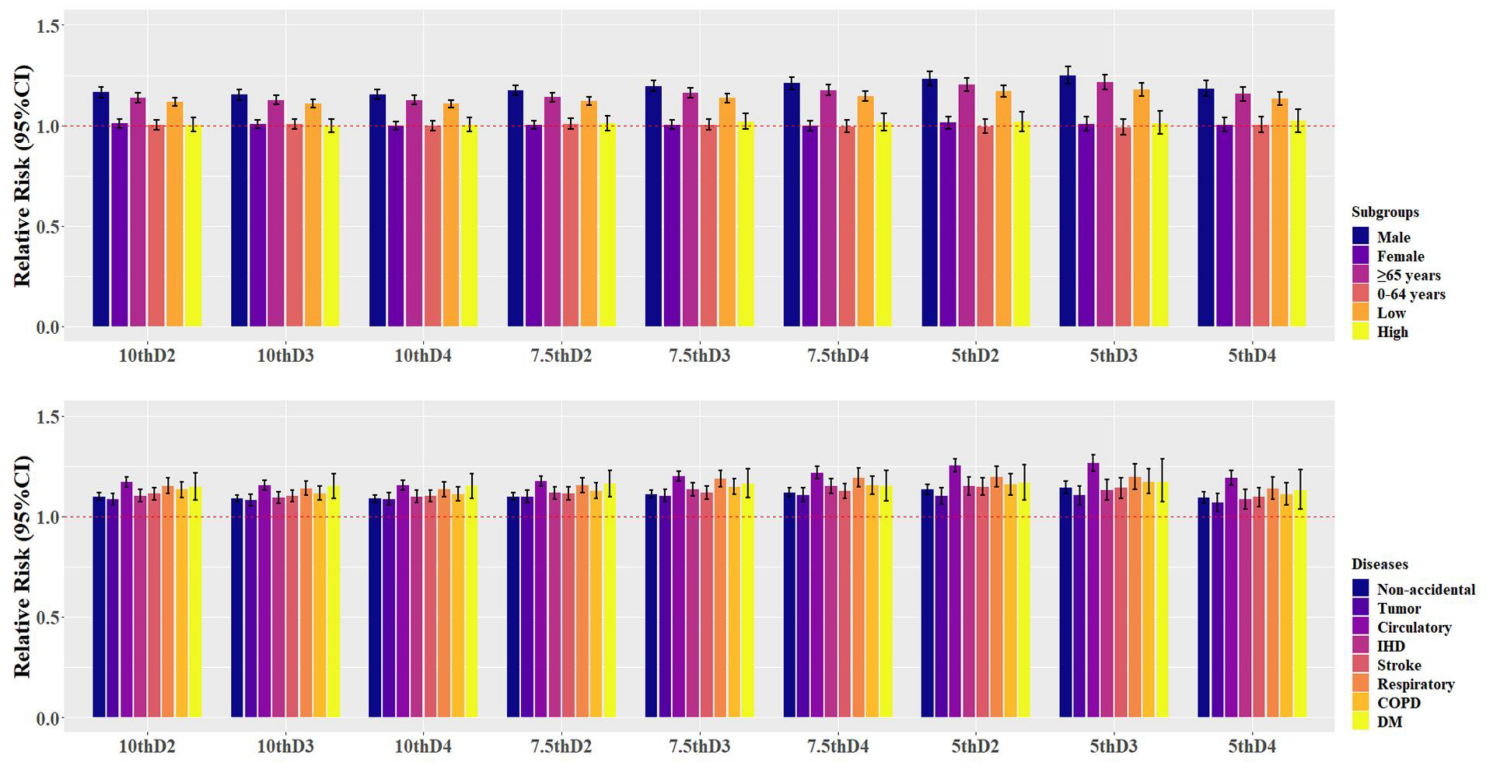


**Figure A5** Single-day lagged effects of the optimal cold spell on different specific causes of death across the lags of 0–21 days. (Non-accidental, Circulatory disease, IHD, Stroke, Respiratory disease, COPD, Tumor, and Diabetes mellitus)


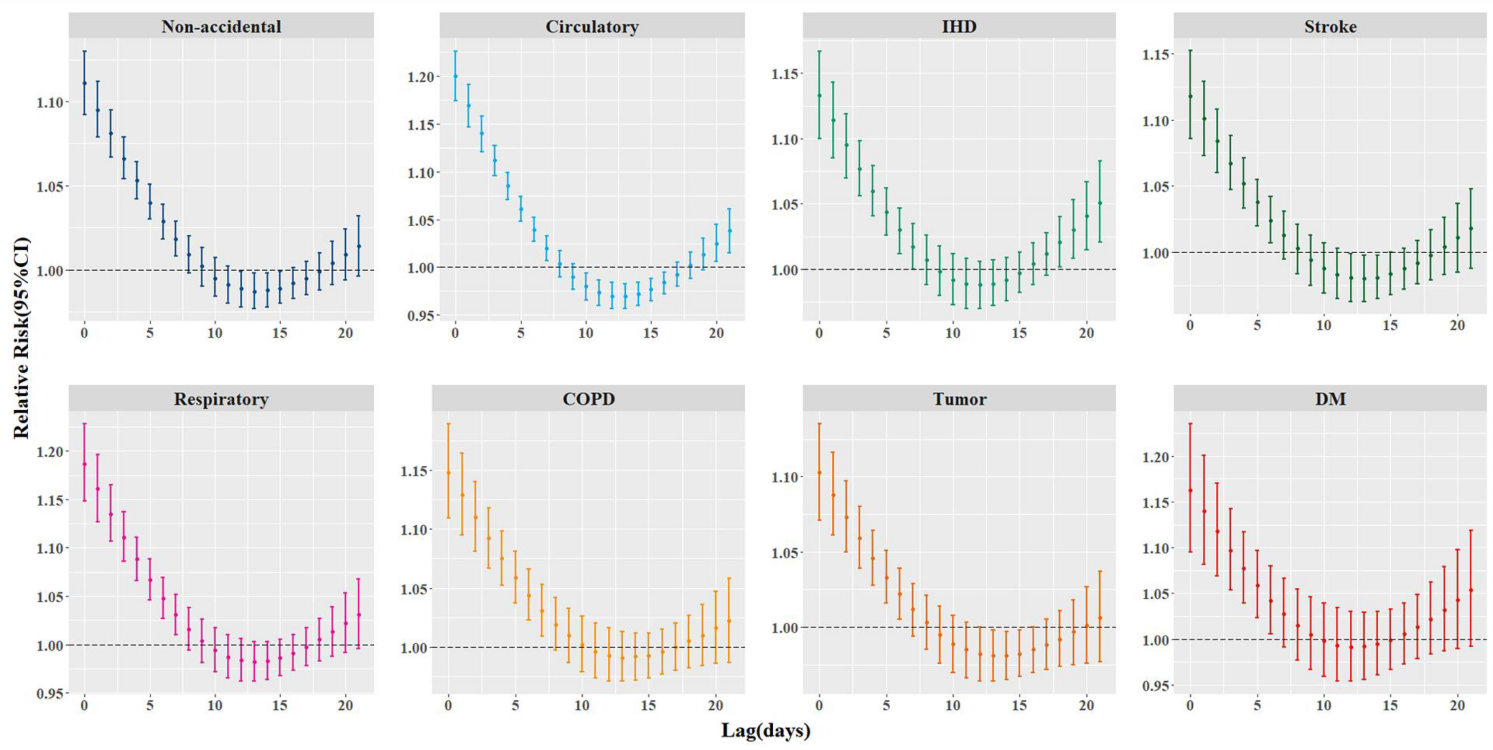


**Figure A6** The cumulative lagged effects of the cold spell on specific causes of death for different residents over two periods, from 2016 to 2018 and from 2019 to 2020.


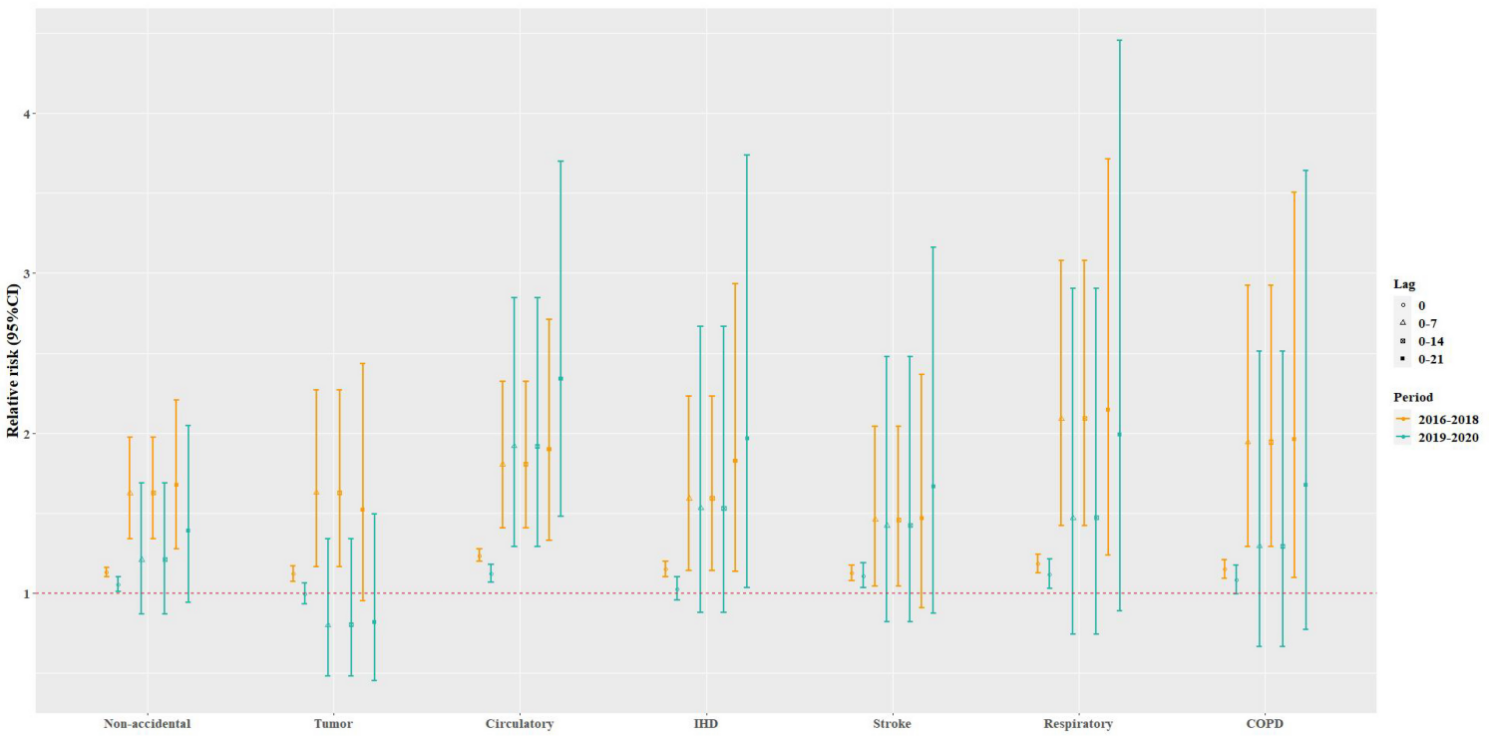


**Figure A7** Sensitivity analyses for estimates of cold spells on non-accidental mortality.


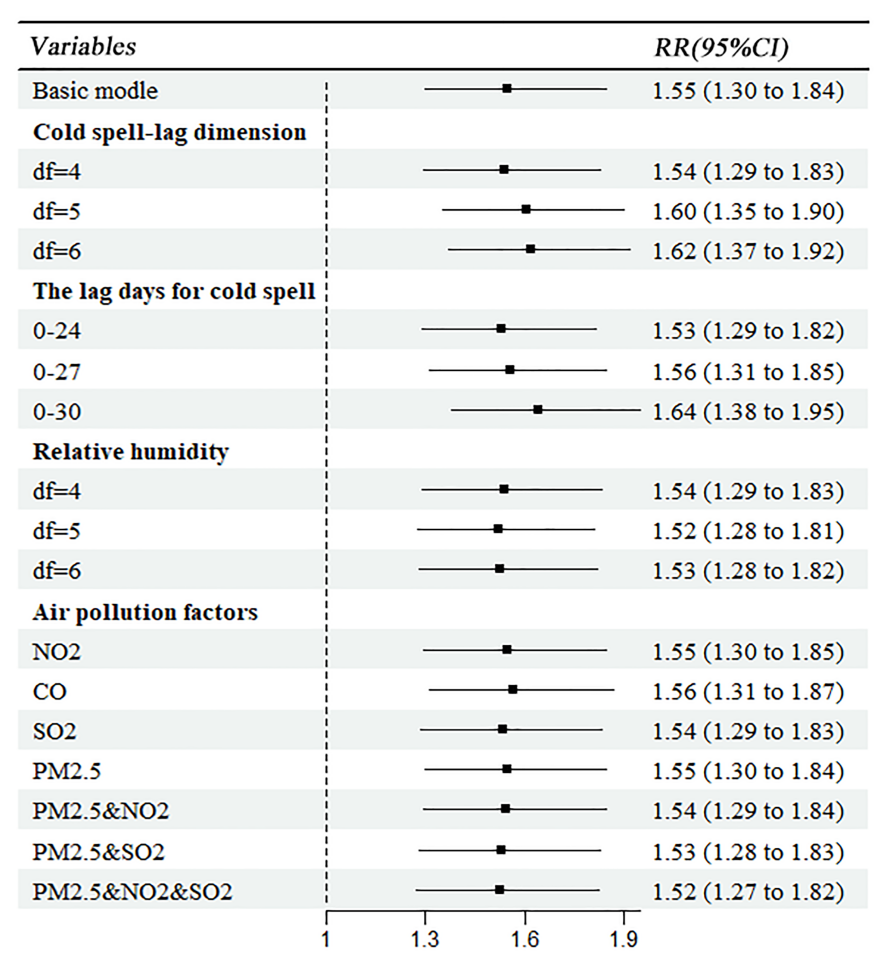

Supplement: Supplementary file 1 — Supplementary Materials1. [file 13690_2024_1284_MOESM1_ESM.docx]
